# Progress in the Preparation of Stimulus-Responsive Cellulose Hydrogels and Their Application in Slow-Release Fertilizers

**DOI:** 10.3390/polym15173643

**Published:** 2023-09-04

**Authors:** Zhenghui Li, Ming Zhang

**Affiliations:** 1 School of Material Science and Engineering, Beihua University, Jilin City 132013, China; lizhenghui202304@163.com; 2 Key Laboratory of Wooden Materials Science and Engineering of Jilin Province, Beihua University, Jilin City 132013, China

**Keywords:** slow-release fertilizers, hydrogel, cellulose, stimulus-responsive, release mechanism

## Abstract

Agriculture is facing challenges such as water scarcity, low fertilizer utilization, food security and environmental sustainability. Therefore, the development of slow-release fertilizer (SRF) with controlled water retention and release is particularly important. Slow-release fertilizer hydrogel (SRFH) has a three-dimensional (3D) network structure combined with fertilizer processing, displaying excellent hydrophilicity, biocompatibility and controllability. Cellulose has abundant hydroxyl groups as well as outstanding biodegradability and special mechanical properties, which make it a potential candidate material for the fabrication of hydrogels. This work would analyze and discuss various methods for preparing stimulus-responsive cellulose hydrogels and their combinations with different fertilizers. Moreover, the application and release mechanism of stimulus-responsive cellulose hydrogels in SRF have been summarized as well. Finally, we would explore the potential issues of stimulus-responsive cellulose hydrogels serving as an SRF, propose reasonable solutions and give an outlook of the future research directions.

## 1. Introduction

Since the global population is increasing and food shortage issues are becoming more and more serious, fast-acting fertilizers such as nitrogen (N) phosphorus (P) and potassium (K) are essential in modern agriculture to improve food production. In the last 50 years, global agriculture has been improved due to the introduction of N fertilizers. The current data show that 2.50 × 10^6^ tons of fertilizers will be needed annually to meet global food demand, an 800% increase in N fertilizer inputs, 35% of which has been used in China [1]. However, 50–70% of N fertilizer is usually lost to the environment through volatilization, denitrification and leaching. Instead, further overuse of N fertilizer is not effective in improving crop yields, but will increase the nitrate and N_2_O (greenhouse gases) levels in groundwater [2], leading to severe environmental pollution and low fertilizer efficiency [3]. Nowadays, almost 87% of the globally consumed water was applied in irrigating the lands to increasing yield, the contribution of which has reached more than 40% [4]. However, the high evaporation, low rainfall, high variability, uneven seasonal distribution, high frequency of droughts, and serious water scarcity in arid and semiarid regions greatly limit the cultivation and production of crops [5]. Apparently, the fertilizer and water resources are not only the two main factors affecting crop growth, but also the key factors for sustainable agricultural development [6]. Therefore, it is of great importance to develop a novel fertilizer with good water-holding and slow-release properties. Slow-release fertilizer hydrogel not only can retain more water in the soil, but can also meet the various nutrient requirements towards the crops at different growth stages.

Due to the porous structure and intermolecular interactions, hydrogel is able to provide the loading sites for fertilizers. Its 3D hygroscopic network structure has excellent water absorption and retention capacities, but it is insoluble in water, and can retain large amounts of water/liquid fertilizer for longer periods of time, which plays amazing roles in SRF by slowly releasing in stressful environments. Stimulus-responsive hydrogels can sense a little change or stimulus from the external environment (e.g., temperature, pH, light, etc.), and subsequently undergo variations in physical structure or chemical properties [7]. After embedding fertilizers into stimulus-responsive hydrogels through dissolution adsorption, in situ embedding and coating envelopes, the as-obtained SRF can change its release behavior according to the variations of surrounding environment and the fluctuations in soil. It can provide a more efficient path for fertilizer delivery, which facilitates the proper amount of fertilizer applied at the optimal time, and synchronizes with the plant’s nutrient requirement more precisely [8]. Despite the above attractiveness, the hydrogels have some drawbacks, such as the following: some physical hydrogels are soft and fragile (with weak mechanical properties, e.g., strength~100 kPa, rigidity~10 kPa and toughness~10 J/m^−2^) [9], which are hardly expected to retain their shape and robustness; although the petroleum-based hydrogels represented by polyacrylamide (PAM) and polyacrylic acid have high water absorption and retention, they are expensive, poor biodegradability, and have potential environmental hazards (e.g., their degradation products lead to the function disorders of normal soil), which are not conducive to the sustainable development of agriculture [10]. Therefore, the incorporations of natural polymers and nanomaterials (e.g., cellulose nanofiber, etc.) into the matrix not only enhance the mechanical properties of the hydrogels, but also solves the high-cost and low-degradability problems. 

Nowadays, the green, biodegradable and renewable polymers (e.g., cellulose [11], lignin [12], starch [13], chitosan [14], etc.) have been used to prepare SRF under the “carbon peaking and carbon neutrality” strategy. They greatly reduce the production costs, simplify the manufacturing process, and solve the environmental problems caused by excess by-products. Some factors should be considered during the design procedure as follows: (a) to load sufficient nutrients; (b) the safety, efficiency and biodegradability of designed material; (c) no interference towards vegetation germination [15]; and (d) to release the fertilizer according to the stimulation of plant growth stages [3]. As a polymeric compound widely found in plant cell walls, cellulose has been considered as the most abundant and renewable material on earth [16]. It contains abundant functional groups, exceptional mechanical properties and high chemical modifiability. Moreover, cellulose could be produced into fibrous forms at the micro/nanometer size scale, which becomes a promising candidate for the fabrication of hydrogels in recent years [17,18,19,20,21]. Cellulose-based hydrogels can be prepared by physically crosslinking the cellulose molecules, or by chemically and/or physically crosslinking the cellulose derivatives [22]. The attractive properties of cellulose and its derivatives (e.g., ethylcellulose, cellulose acetate, methylcellulose, etc.) contain properties of biodegradability, biocompatibility, nontoxicity and functionality, which have motivated scientists and researchers worldwide to develop the cellulose hydrogels for a variety of applications. Notably, cellulose also improves the mechanical strength and biodegradability of the composite hydrogel product, which has high potential as an agricultural-grade reinforcing material in the manufacture of environmentally friendly stimulus-responsive hydrogel, indicating its promising application in SRF [23]. 

**Figure 1:**
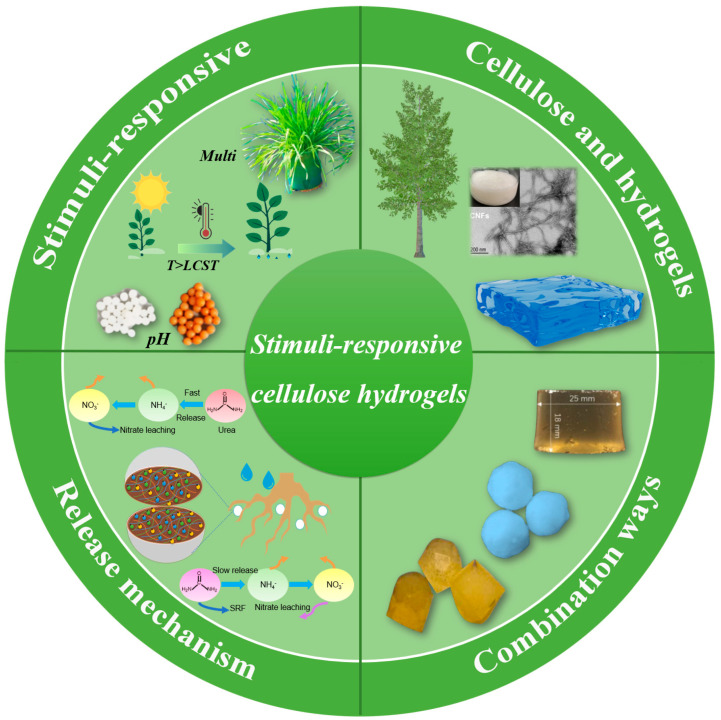
Schematic diagram showing the main topics of this review.

To control the release of cellulose hydrogels serving as the SRF, an in-depth understanding of the slow-release mechanism is necessary for further research and following applications [24]. The release mechanism of cellulose hydrogel is a hot research topic, and many reasonable release models have been proposed by many scholars. However, its release mechanism of nutrients cannot be determined uniquely due to the influence of original materials, soil and other factors [25,26,27]. This work would introduce and analyze various methods for preparing stimulus-responsive cellulose hydrogels and their combinations with different fertilizers. From Figure 1 and Table 1, the application and release mechanism of stimulus-responsive cellulose hydrogels in SRF would be discussed as well. Moreover, the potential issues of stimulus-responsive cellulose hydrogels serving as the SRF would be summarized. Finally, we also propose the reasonable solutions and outlook the future research directions of stimulus-responsive cellulose hydrogels in SRF.

## 2. Preparation of Hydrogels Based on Cellulose and Its Derivatives

### 2.1. Cellulose and Its Derivatives

Cellulose, first discovered and isolated by Anselme Payen in 1838, is a mixture of crystalline and amorphous forms consisting of glucose units linked by β-(1,4) glycosidic bonds, which play an important role in maintaining the structure of plant cell walls. As a natural polymer, cellulose is resourceful, biocompatible, renewable and degradable, which is why it is used in various applications such as food packaging [32], electromagnetic shielding [33], drug carriers [34], 3D printing [35], lithium–sulfur and zinc batteries [36,37], etc. Cellulose with an annual production of over 7.5~10.0 × 10^10^ tons is distributed in higher plants and marine animals (e.g., tuna) widely, and in algae, fungi, bacteria, invertebrates and even amoebae (protozoa) to a lesser extent [38]. In general, cellulose is a fibrous, tough, water-insoluble substance with the advantages of high mechanical strength, high Young’s modulus, large specific surface area, hydrophilicity, and chemical modification potential, which has become a potential candidate for manufacturing cellulose-based hydrogels in recent years [39]. 

**Figure 2:**
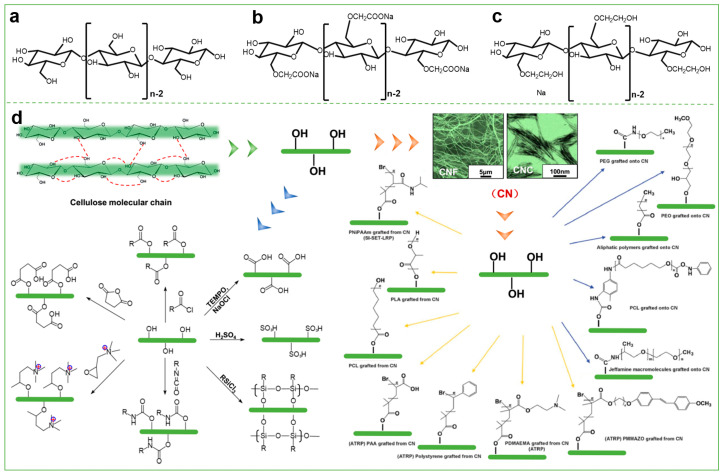
Chemical structures of (**a**) cellulose, (**b**) carboxymethyl cellulose (CMC), and (**c**) hydroxyethyl cellulose (HEC). (**d**) Schematic illustration of modification and functionalization of cellulose and its derivatives.

At the nanoscale level (≤100 nm), nanocellulose is a class of flexible and elongated nanostructured cellulose, which can be usually separated from wood and other plants by specific pretreatments (e.g., deep eutectic solvent, carboxymethylation, TEMPO oxidation, homogenization, defibrillation, etc.) and the mechanical shearing procedure [10]. The chemical and physical features of nanocellulose usually vary depending on its source and extraction method (e.g., physical, chemical and biological methods, etc.). Nanocellulose fibers possess the merits of natural cellulose such as low density, nontoxicity and biodegradability, as well as high mechanical strength, high crystallinity, high specific surface area and self-assembly in aqueous media due to their nanoscale shape and size [40]. According to size and structure characteristics, nanocellulose is usually classified into cellulose nanocrystals (CNCs) [41], cellulose nanofibrils (CNFs) [42], and bacterial cellulose (BC) [43]. Notably, the intermolecular and intramolecular hydrogen bonds in cellulose and its high crystallinity greatly limit its water solubility and reactivity as well as its respective application. For the effective utilization of cellulose, a series of cellulose derivatives have been obtained after modification and functionalization through the chemical reactions (e.g., amination [3], esterification [44], etherification [45], oxidation [46], etc.) of the hydroxyl groups in the cellulose molecule as presented in Figure 2. These derivatives (e.g., cellulose ethers, cellulose esters and cellulose phosphates, etc.) have been widely used in material science, biomedicine and other fields. 

Cellulose ethers, as common cellulose derivatives including carboxymethyl cellulose (CMC), hydroxyethyl cellulose (HEC) and hydroxypropyl cellulose (HPMC), are the compounds obtained by reacting cellulose with alcohols, phenols, and amines through alkylation and etherification processes. They have good dispersion, water retention, and emulsification and thickening properties, which are commonly used in food, cosmetics, pharmaceuticals, building materials, etc. [47]. CMC is mainly used as a thickener, suspending aid, binder, gelling agent, stabilizer, water-retention agent, etc., displaying an outstanding ability to stabilize emulsions, absorb moisture from the atmosphere, and suspend solids in aqueous media. Sanosh et al. [48] obtained a sodium carboxymethylcellulose (CMC) hydrogel from the etherification between BC with chloroacetic acid in an alkaline medium. The as-prepared CMC without stirring (CMC-0 h) after dehydrothermal treatment generated the crosslinked hydrogel, which has a water absorption of 35 times its original weight. This work paves the path to artificial ultrapure hydrogels for their use in the healthcare and pharmaceutical industries. In an acidic environment, the hydroxyl group of cellulose can be esterified with the acyl halides and acid anhydrides to produce the cellulose esters with good solubility and film-formation properties. Cellulose esters have been commonly used in coatings, plastics, films, etc. Cellulose phosphate is a compound obtained by the reaction of cellulose and phosphoric acid. Due to its excellent flame retardancy and biocompatibility, cellulose phosphate has been widely used in textiles, medical materials, and other fields [49].

In addition, enolic acid, amide, olefin and other molecules containing unsaturated bonds can be used to modify cellulose’s molecular structure for obtaining the cellulose derivatives with various functional groups by the grafting reaction. These cellulose derivatives not only retain the green and environmental advantages of natural cellulose, but also provide a large number of functional groups such as hydroxyl, carboxyl and amino groups, which contribute to the preparation of hydrogels with 3D porous structure, and endow them with new functional properties and application values. Furthermore, numerous hydroxyl groups in nanocellulose can adsorb the charged contaminants [50,51]. The products designed based on nanocellulose display many amazing properties such as excellent mechanical properties, good thermal stability, 3D porous structure, strong adsorption capacity towards metal ions and dyes, being an ideal carrier for loading nutrients, being fluorescent probes and other functional molecular properties [50,52]. 

### 2.2. Hydrogel and Its Crosslinking Strategy

Hydrogel is made from the combination of synthetic or/and natural polymers. As a polymeric material with a highly porous 3D network structure and a large number of hydrophilic groups (e.g., amines, hydroxyl and carboxylic) [53], hydrogel can absorb and hold substantial water or biological fluids (higher than 20%) without disintegration. In 1960, Wichterle and Lim co-polymerized glycolmonomothacrylate and glycoldimethacrylate to achieve the first generation of hydrogel, which greatly improved the compatibility between the materials and live tissues. Due to the outstanding biocompatibility, physical and chemical properties, hydrogel rapidly triggered a research boom in scientific fields since then, which has been widely used in tissue engineering [54], controlled drug release [55], agricultural production [56], hygiene products [57], and electronic devices [58]. Moreover, hydrogels have high permeability for water-soluble drugs, tunable mechanical properties, predictable degradation rates, and sensitivity to external stimuli such as pH and temperature [59].

The physical and chemical properties of hydrogels (swelling property, mechanical strength, elasticity, release kinetics, etc.) highly depend on their composition, stimulus condition (e.g., pH, temperature, light, magnetic field, etc.), nature and extent of crosslinking [60]. Physical crosslinked hydrogels, also called non-permanent hydrogels, are caused by macromolecular entanglement, ion attraction, electrostatic interaction, hydrogen bonds, van der Waals forces and hydrophobic interactions. Physical hydrogels are usually reversible (sol–gel transition) and preferable for applications where toxic crosslinkers are undesirable, and are often formed under modest conditions [16]. Although they have the advantages of easy preparation and operation, they also have the disadvantages of low mechanical strength and poor stability [61]. Common crosslinked hydrogels prepared via physical strategy include freeze-dried hydrogels (freeze-drying technology) [62], filament hydrogels (self-assembly and other methods) [63] and nanogels (nanoparticle formation) [64]. In conclusion, physical hydrogels have diverse structures, but poor mechanical properties and can easily be lost in soil, which are unfavorable to water retention and the slow release of fertilizer, greatly limiting their application in agriculture.

Chemical crosslinked hydrogels, also known as permanent hydrogels, are thermally irreversible (differ from physical crosslinked hydrogels), which are more stable in soil serving as the SRF for agriculture due to its rigid networks, mechanical robustness, high water absorption, and excellent physical and viscoelastic properties [16,65]. Usually, chemical crosslinked hydrogels are achieved by forming covalent bonds among the polymer chains in the presence of crosslinking agents or under specific external conditions. Such procedures might involve covalent bond graft, free-radical polymerization, click chemistry, enzymatic reactions, heat dehydration, and ionic and radiation (e.g., gamma and ultraviolet rays, or electron beam) [3,15,31,59] crosslinking [66,67,68,69,70]. Common chemical crosslinked hydrogels include PAM hydrogels [71], polyvinyl alcohol hydrogels [72], acrylic hydrogels [73] and so on. Although chemical crosslinked hydrogel has the mature preparing techniques and wide range of applications, its crosslinking process is usually tedious, and the introduced crosslinking agent may be toxic [74]. The chemical hydrogels synthesized via radiation without toxic crosslinking agent are relatively pure and safe, which are suitable for agriculture [70], biomedical and pharmaceutical applications [75]. 

Dual-network crosslinked hydrogels are formed by interweaving two identical or different networks, which are generally realized by free-radical copolymerization, water-soluble polymer crosslinking, and polymer interpenetrating networks [76]. One network is strongly covalently crosslinked (via covalent bond graft, free-radical polymerization, click chemistry, heat dehydration, etc.), which usually has excellent mechanical strength and rigidity [77]. Meanwhile, the other one is loosely physically crosslinked (via van der Waals forces, electrostatic interactions, hydrogen bonding, etc.), which usually has high ductility and reversibility [78]. These two networks can be entangled with each other by physical or chemical crosslinking. The hydrogel with dual networks is obviously superior to the single network hydrogel, showing excellent ductility, high plasticity, good biocompatibility, and outstanding self-healing property, which can withstand large tensile and compressive forces [79]. However, the radiation and dual-network crosslinking processes are usually complicated and expensive, which should be further simplified and lower the cost. Therefore, it is significant to develop a simple and low-cost process for preparing the water-retaining, structurally stable, biodegradable and nontoxic crosslinked hydrogels by using natural biomass materials.

### 2.3. Hydrogel Based on Cellulose and Its Derivatives

The mechanical properties of hydrogels are vital to preserving the shape, stiffness, and robustness of the hydrogel during its practical applications [54]. However, many pure hydrogels are very soft, displaying a weak mechanical property. The hydrogels based on cellulose and its derivatives, so-called cellulose hydrogels, can be obtained by the esterification or etherification of cellulose, and the chemical or dual-network crosslinking with cellulose derivatives [16]. Due to their extraordinary mechanical properties, high absorption efficiency and stimulation reactivity, the cellulose hydrogels are increasingly attractive in multidisciplinary fields (e.g., biomedicine [34], food [80] and agriculture [81]) [16]. 

Due to its high strength, crystallinity, surface area and aspect ratio, nanocellulose (e.g., CNF and CNC) has attracted increasing attention in the preparation of nanocomposite hydrogel as the reinforcing filler. Moreover, cellulose nanocomposite hydrogel can be more qualified to serve as the reservoir for improving irrigation efficiency and plant growth. Xu et al. [82] reported a novel nanocellulose hydrogel based on TEMPO-oxidized CNF and gelatin methacrylate (GelMA) for 3D printing the scaffold. The product can uptake water up to around 90 times its own weight. The hydrogel scaffold with high CNF concentration after dual crosslinking treatment achieved a further improvement of its mechanical strength. Its compressive Young’s modulus and local surface stiffness could be adjusted by changing the CNF and GelMA contents. Specifically, the introduction of nanocellulose and its derivatives not only improves the mechanical strength of the hydrogel, but also enhances its biodegradable capability, which is conducive to green, environmentally friendly and sustainable development.

**Figure 3:**
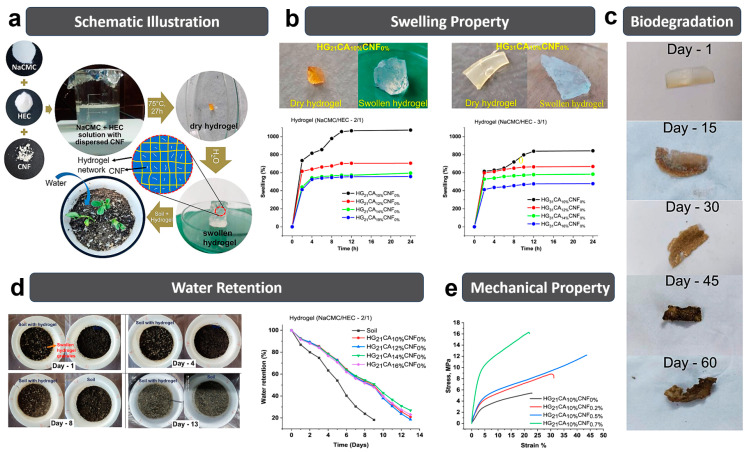
(**a**) Schematic illustration of NaCMC/HEC/CNF hydrogels. (**b**) Swelling performance of hydrogels: NaCMC/HEC:2/1 and NaCMC/HEC:3/1. (**c**) Hydrogel images during the buried soil test. (**d**) Water retention test of soil with and without hydrogel. (**e**) Stress−strain curves of the hydrogel and hydrogel nanocomposites. Reprinted with permission from ref. [83]. Copyright 2023 American Chemical Society.

Das et al. [83] synthesized the hydrogel based on the carboxymethyl cellulose sodium (NaCMC), HEC and CNF with citric acid (CA) as the crosslinking agent (Figure 3a). The HEC greatly promoted the interaction between the molecular chains for forming a strong hydrogel network, and the CNFs (with a length of ~600 nm, 0.7%) served as the reinforcing agents in the hydrogel matrix, which largely improved the mechanical strength of hydrogel (16.27 MPa, Figure 3e). Such a mechanical improvement is essential to maintain the structural integrity of the hydrogel in agricultural applications. The hydrophilic groups (e.g., −OH and −COOH) attached to the cellulose chains are responsible for the water-absorbing capability of hydrogel. The water absorption of NaCMC/HEC/CNF hydrogel could reach up to ∼1070% (Figure 3b,d). Moreover, the buried soil tests (60 days) showed appearance and color variations, structural decomposition and chemical changes of the hydrogels due to microorganism degradation (Figure 3c), confirming its outstanding biodegrading capability.

In summary, hydrogel based on cellulose (or nanocellulose) and its derivatives can be prepared by different crosslinking methods. Its outstanding overall performance makes it fit in absorbing and retaining water in the hydrogel networks, and releasing water and nutrients to the plants in a controlled manner, which is very essential for agricultural production in arid and semiarid regions.

## 3. Combination of Fertilizer and Cellulose Hydrogel

In the growth of crops, nitrogen, phosphorus, potassium, and some other trace elements (such as iron, zinc and molybdenum) are the essential nutrients [84]. The pore structure of cellulose hydrogels can provide suitable sites for loading the urea [85]. It is the recent research focus to combine cellulose hydrogel with nutrient fertilizer for obtaining a novel hydrogel with slow-release function. This paper summarizes three main strategies for loading the fertilizer into cellulose hydrogel in the following section.

### 3.1. Solubilization and Adsorption Method

**Figure 4:**
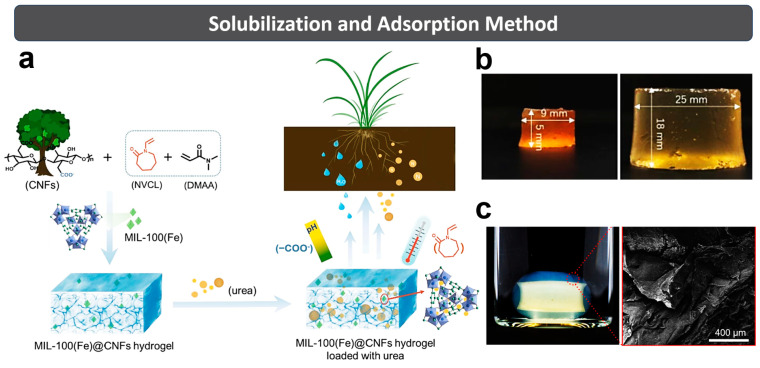
(**a**) Simplified scheme for preparing MC hydrogel. (**b**) Hydrogels before and after swelling equilibrium. Reprinted with permission from ref. [31]. Copyright 2021 Elsevier Ltd. (**c**) Alginate-coated gelatin/CNC hydrogel and its SEM image. Reprinted with permission from ref. [87]. Copyright 2022 Elsevier Ltd.

Generally, the solubilization and adsorption method involves three steps (Figure 4). The first step is immersing the as-prepared aerogel (freeze-dried hydrogel) in a solution containing fertilizer. The second step is the adsorption of nutrients into the 3D network by the swelling of hydrogel. The third step is waiting for the equilibrium swelling ratio (ESR) of hydrogel with ultimate nutrient and water retention. In addition, the releasing speed of the hydrogel can be controlled by changing its physical shape after drying [86].

Liu et al. [31] used MIL-100(Fe) (metal–organic frameworks, MOF), CNF and sodium alginate (SA) to prepare the MC hydrogels by free-radical polymerization in the presence of APS (initiator) and MBA (crosslinking agent). Subsequently, the resulting hydrogel was dried using a freeze dryer, and further immersed into the urea solution (25 mg/mL) for 72 h at 25 °C, as presented in Figure 4a. Results of the fertilizer slow-releasing test show that the cumulative release rate of MC-10% (MOF to CNF: 10%) hydrogel after loading urea reached 40.84% in soil at 30 d.

Do et al. [87] prepared an alginate-coated gelatin/CNC hydrogel by a simple layer-by-layer process (Figure 4c). Then, NH_4_NO_3_, P_2_O_5_ and K_2_O (NPK fertilizer) were dissolved in the distilled water to prepare NPK aqueous solution (10 mg·mL^−1^). After immersing in NPK aqueous solution (100 mL), the pre-dried hydrogels gradually swelled to a steady state. Results showed that the alginate-coated gelatin/CNC hydrogel exhibited a slower NPK releasing speed than the gelatin/CNC hydrogel. Compression tests showed that the addition of CNCs improved the mechanical properties, water-retention capacity, and sustained fertilizer release of the hydrogel.

In summary, the solubilization and adsorption method can reduce the fertilizer’s influence on the hydrogel-synthesis process, and better control the formation of SRFHs. However, the fertilizer loading in hydrogel is lower than the other methods, and the multifarious operation steps (including hydrogel synthesis, drying, fertilizer absorption and re-drying) leads to a long preparation period [65]. Moreover, the hydrogel must be crosslinked sufficiently to envelop the fertilizer very well.

### 3.2. In Situ Encapsulation Method

**Figure 5:**
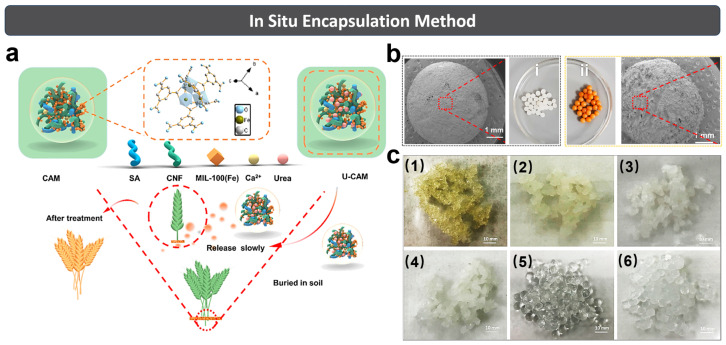
(**a**) Simplified scheme for preparing the CAM hydrogel and its application in the slow release of urea. (**b**) SEM and photograph of (i) CAM-0% (without MIL-100 (Fe)); (ii) CAM-10% (MIL-100 (Fe) to CNF at 10%). Reprinted with permission from ref. [15]. Copyright 2021 Elsevier Ltd. (**c**) Different hydrogels after swelling for 200 h. (1) SA, (2) SA/NPK, (3) SA/CNF/NPK, (4) SA/CNF, (5) SA/CNF/PVA and (6) SA/CNF/PVA/NPK hydrogels. Reprinted with permission from ref. [28]. Copyright 2021 Elsevier Ltd.

The in situ encapsulation method (Figure 5) involves directly mixing the fertilizer and raw materials together. During the formation of hydrogel, the fertilizer can be encapsulated in the hydrogel matrix [65]. Wang et al. [15] prepared the CNF from pulp residue, which was further mixed with SA and urea in the presence MIL-100 (Fe) under stirring condition. Then, the ball-shaped CNF/SA/MOFs hydrogel (CAM) with good water- and fertilizer-regulating functions was prepared successfully by dropping the above mixture uniformly in CaCl_2_ solution (200 mL, 2.5 wt%) by using a syringe (Figure 5a,b).

Liu et al. [28] prepared the CNF suspension (0.5 wt%), which was further mixed with SA under stirring condition to obtain a homogeneous suspension. Then, polyvinyl alcohol (PVA) and NPK fertilizer (1.5 wt%) were added to the above suspension, which was loaded into two plastic syringes and then pushed into a beaker containing CaSO_4_ solution (400 mL 0.1 wt%). Then, slow-release fertilizer cellulose hydrogels with a macroporous flexible core and microporous semi-interpenetrating polymer network shell were successfully prepared (Figure 5c). Results showed that the water of the soils containing composite hydrogel was beyond 80% on the 60th day, and the N/P/K releases of composite hydrogel was 14.03%/11.95%/7.33% (1st day) and 67.90%/70.78%/71.12% (30th day), respectively.

Compared with the solubilization and adsorption method, the in situ encapsulation method can load more fertilizer into the hydrogel, largely increasing the fertilizer loading capacity of cellulose hydrogel. Furthermore, this method is much simpler than the solubilization and adsorption method, which only requires one step to load the fertilizer in hydrogel, largely reducing the preparing cost and time for SRFH. Notably, the presence of fertilizer sometimes might adversely affect the formation and properties of the hydrogel. Therefore, the polymerization process must be strictly controlled until its completion in order to avoid the unreacted materials and the impurities which are difficult to remove [65].

### 3.3. Coating and Wrapping Method

**Figure 6:**
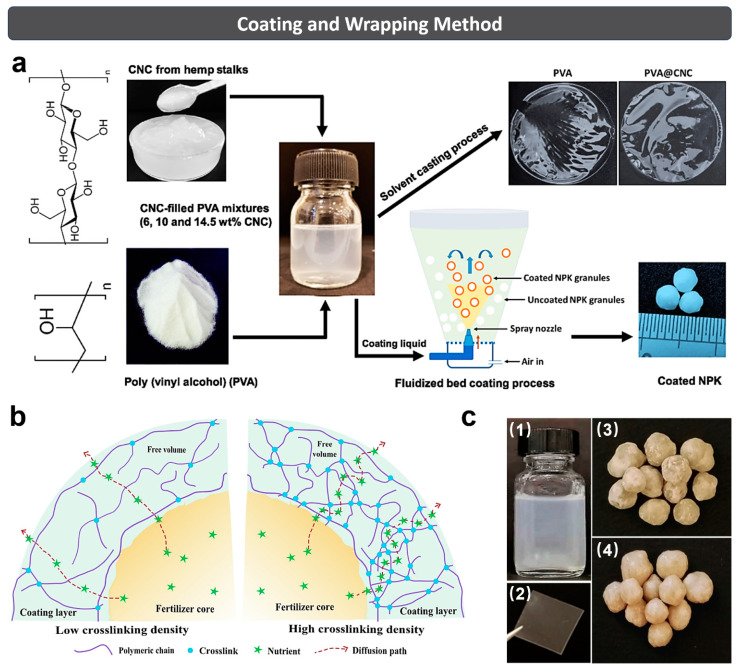
(**a**) Schematic illustration for preparing PVA@CNC nanocomposites and further coating on the NPK fertilizer granules. Reprinted with permission from ref. [88]. Copyright 2021 Elsevier Ltd. (**b**) Schematic illustration of the relationship between the crosslinking degree of coating and the nutrients diffusion of coated MAP fertilizer. (**c**) Photographs of CH@5RC (1) formulation and (2) solid film, (3) uncoated and (4) coated MAP granules. Reprinted with permission from ref. [17]. Copyright 2022 Elsevier Ltd.

Generally, the coating and wrapping method (Figure 6) involves (1) wrapping the fertilizer as a core in a hydrogel with water-absorption and retention capacity, and (2) forming a core–shell structure to achieve the effect of water retention and SRF. The product core can be pure urea or NPK composite fertilizer; in the meantime, some polymers and inorganic substances can be added to regulate its performance as well. The shell can be a water-absorbing and water-retaining hydrogel with one or multiple layers, or a layer of hydrophobic polymer which can delay the release of water and fertilizer.

Kassem et al. [88] reported a waterborne and biodegradable PVA@CNC nanocomposite hydrogel formulation, which was further applied on the surface of NPK fertilizer granules to form the microlayer in the Wurster chamber of a fluidized bed dryer (Figure 6a). It was found that such a coating layer extended the NPK nutrients’ release time from 3 days for uncoated fertilizer to 30 days for PVA@CNC-coated fertilizer in soil medium. Furthermore, the crushing strength of fertilizer was increased after PVA@CNC coating. The improved water-retention capacity of the soil amended with PVA@CNC-coated NPK fertilizer demonstrated the intrinsic water-saving property of PVA@CNC. Apparently, the facile coating method indeed brings physical and chemical advantages to the conventional fertilizer in terms of structural stability, nutrient release and water management.

In 2022, Kassem et al. [17] developed a waterborne and swellable hydrogel formulation as the wrapping material for monoammonium phosphate (MAP) fertilizer. Specifically, sodium CMC/HEC was prepared and composited with regenerated cellulose (RC, 5%) to obtain CH@5RC formulation and solid film (Figure 6b,c). Two coating thicknesses (27 μm, and 71 μm) and two crosslinking conditions (80 °C/8 h, 120 °C/10 min) were utilized to apply the CH@5RC on MAP fertilizer in a spraying process. The relationship between the crosslinking degree of coating and the nutrient diffusion of coated MAP fertilizer were discussed in this study as well. Results showed that the crushing resistance of coated fertilizer reached up to 90.55 N, and the soil containing the coated fertilizer obtained the better water retention. Specifically, the release time of MAP fertilizer can double and better retain the soil moisture when cellulose serves as the main coating material.

To sum up, the coating and wrapping method can greatly reduce the release rate of nutrients by forming a dense coating or wrapping multiple layers with less porosity on the fertilizer surface [65]. Although the process is complex and of high cost, it can integrate various good performances into the product with excellent water retention and a slow/controlled release speed of the fertilizer by wrapping different types of coatings.

## 4. Slow-Release Fertilizer Hydrogels and Release Mechanism

Slow-release fertilizers (SRFs) or controlled-release fertilizers (CRFs) are the nutrients that release into the environment in a slow and controlled manner. There is no official differentiation between CRFs and SRFs. The association of American plant food control officials (AAPFCO) has stated that SRF is a fertilizer that delays its nutrient delivery and availability to plants after application [65]. According to the instructions of the European standards committee, if the nutrient release at 25 °C satisfies the following three conditions, it can be classified as SRF: (1) no more than 15% within 24 h; (2) no more than 75% within 28 d; and (3) at least 75% within the specified time frame. Compared to conventional fertilizers, they have significant advantages in reducing nutrient loss, improving nutrient utilization, increasing crop yields, and protecting the environment. Therefore, the application of SRF brings many advantages: (1) it facilitates the supply of nutrients to plants throughout the growing season, (2) it reduces the labor and energy costs required for multiple fertilizer applications, (3) it improves safety due to the reduction of specific toxicity caused by high ion concentrations in the soil, (4) it reduces nutrient losses from the soil, and (5) it alleviates environmental problems (e.g., eutrophication of water body) [89].

Slow-release fertilizer hydrogels (SRFHs) release the nutrients slowly, thus reducing the evaporation losses and frequent irrigation. They not only lessen the environmental impact of conventional fertilizers, but also enhance plant nutrition. Such an approach offers a high-quality solution to water and fertilizer management, which effectively boosts food production in arid/semiarid regions, promoting the sustainable development of environmental agriculture.

### 4.1. Slow-Release Fertilizer Hydrogels

Based on material origins, SRFHs can be categorized into three groups including natural hydrogels, purely synthetic hydrogels, and synthetic–natural hybrid hydrogels. With the increasing emphasis on environmental protection and green chemistry, natural polymers gained popularity due to their safety, biocompatibility and biodegradability. Natural SRFHs are often fabricated by natural polysaccharides such as cellulose, starch, chitosan, alginate and so on [85]. Purely synthetic hydrogels are typically synthesized from hydrophilic monomers or polymers, which are derived from petroleum-based sources, including polyacrylamide (PAAm), polyacrylic acid (PAAc), and the copolymers combining PAAm and PAAc [65]. Despite their advantageous water absorption and water retention, purely synthetic hydrogels still result in significant environmental issues due to their poor biodegradability, and the potential for degradation products to disrupt normal soil function [65,71,73].

By combining the characteristics above, the synthetic–natural SRFHs are developed, which are mainly categorized as organic–inorganic and organic–organic composites [65]. Organic–inorganic SRFHs are created by incorporating the synthetic polymers with inorganic materials. The inorganic components in hydrogels not only reduces the overall costs, but also enhances the properties such as degradability, swelling capacity, barrier performance, mechanical strength, and thermal stability. Common inorganic materials include clay minerals such as attapulgite [90], montmorillonite [91], bentonite [92] and kaolin [93]. These materials can be incorporated into the hydrogels in significant proportions, simultaneously reducing the cost in production and enhancing the swelling capability of hydrogels. Organic–organic SRFHs are crafted by combining the synthetic polymers with natural polymers or fibers. Natural polymers are abundant in nature, which offer many advantages such as mitigating environmental pollution, lowering hydrogel production costs, and enhancing biodegradability and mechanical properties [94].

Usually, agricultural residues are either incinerated or discarded, contributing to environmental pollution and resource wastage. Natural fibers can be derived from these agricultural residues. By grafting or blending the hydrogels with such natural fibers or polymers (e.g., cellulose), it is possible to simultaneously achieve waste utilization, cost reduction, biodegradability, eco-friendliness, and nontoxicity. Moreover, the incorporated fibers can serve as the structural support for enhancing the mechanical strength, water-absorption capacity, and plant growth performance [95]. In summary, the organic components offer elasticity, low density, malleability and toughness, while the inorganic components provide hardness, rigidity and thermal stability.

### 4.2. Release Mechanism of Slow-Release Fertilizers Hydrogels

Further comprehension of the slow-release mechanism is essential to offer theoretical support for the advancement and implementation of SRF. Since there are variations in nutrient-release mechanisms among different types of SRFs, and a complex interaction of factors (composition, soil moisture, temperature, etc.), the release mechanisms of SRFHs cannot be unilaterally defined. Generally, there are mainly four mathematical models used to explore the release mechanism, including the zero-order, first-order, Korsmeyer–Peppas, and Higuchi kinetic models (Table 1). Specifically, the parameter *M_t_/M_∞_* represents the percentage of fertilizer released from the hydrogel at a specific time *t*. The constants *k*_0_, *k*_1_, *k_H_* and *k* correspond to the release constants of the zero-order, first-order, Higuchi, and Korsmeyer–Peppas models, respectively. The release mechanisms are categorized based on the diffusion index (*n*) as follows: (1) Fickian diffusion mechanism: *n* < 0.43; (2) Non-Fickian diffusion mechanism: 0.43 < *n* < 0.85; (3) Case-II transport mechanism: *n* > 0.85.

**Figure 7:**
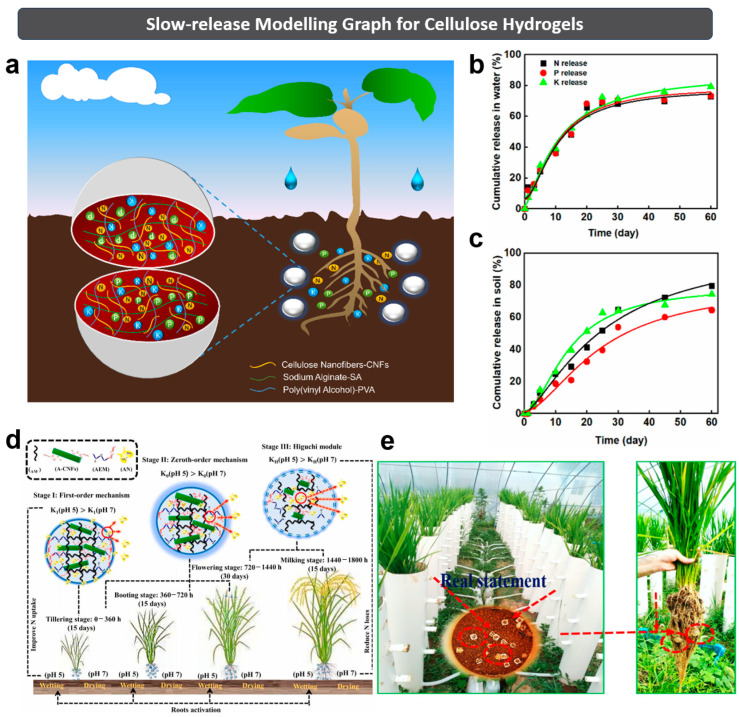
Fertilizer hydrogel: (**a**) schematic diagram, and its NPK release (**b**) in deionized water and (**c**) in soil. The loading rates of SA, CNF, PVA and NPK are 1.5, 0.5, 0.5 and 1.5 wt%, respectively. Reprinted with permission from ref. [28]. Copyright 2021 Elsevier Ltd. (**d**) The relationship between the different rice growth stages and the different AN release stages of fertilizer hydrogel, which are governed by first-order, zeroth-order and Higuchi mechanisms. (**e**) Fertilizer hydrogel in greenhouse pot. Reprinted with permission from ref. [3]. Copyright 2022 Elsevier Ltd.

To date, many efforts have made on the reasonable release mechanisms of SRFHs. Liu et al. [28] prepared a novel SRFH with a macroporous flexible core and a microporous interpenetrating polymer networks (IPN) shell by blending and crosslinking SA, PVA and CNFs in the fertilizer formulations containing NPK (Figure 7a–c). To understand its slow-release mechanism, the authors employed the Korsmeyer–Peppas release kinetic model. The results suggest that the releases of the SA/CNF/NPK and SA/CNF/PVA/NPK hydrogels in water and soil were controlled by Fickian diffusion. However, the release of SA/NPK hydrogel is controlled by erosion as well as Super-Case II transportation. Based on the Korsmeyer–Peppas model, this research group hypothesized the release mechanism of SA/CNF-based hydrogels in the medium (water or soil) as follows.

Firstly, the freeze-dried SA/CNF-based gel slowly swelled due to the water absorption, and converted into hydrogel in the medium. Subsequently, the NPK fertilizer encapsulated in the hydrogel was slowly dissolved. Thirdly, the fertilizer was gradually released into the medium through the dynamic exchange (or diffusion control) between the interior and exterior of the hydrogel. As the swelling ratio increased, the size of the macropores within the 3D network expanded, facilitating the diffusion of the fertilizer into the medium. Finally, the release rate of fertilizer into the medium gradually decreased, and approached a constant value over time. This result indicates that the prepared hydrogel has the potential to be applied in regions with drought-prone conditions or with fertilizer-loss issues, offering possibilities for future applications in precision agriculture and horticulture.

Shaghaleh et al. [3] develop a viewpoint that the practical slow-release model of SRFHs varies at different stages. This research group prepared an aminated-CNF (A-CNF) fertilizer hydrogel by encapsulating ammonium nitrate (AN) in A-CNF hydrogel. According to the literature, its entire duration of sustained AN release was divided into three stages. This division was based on the quantitative analysis of the changes in relative slopes of AN release. Subsequently, the data were fitted with a typical model for each stage (Figure 7d). In the first stage, the fertilizer hydrogel exhibited the fastest AN release within 72 h of incubation. This release behavior was governed by the first-order model (*k*_1_ = 0.068–0.0575). In this stage, A-CNF in the fertilizer hydrogel triggered the swelling procedure subsequent to incubation within the buffer or soil mediums. Consequently, a portion of AN in the hydrogel network undergoes the diffusion, which is influenced by the pH level. Notably, such diffusion predominantly happened from the swollen region surface to the inter-fiber space and the surrounding medium. During the incubation, the swelling process persists. The diameter of A-CNF progressively enlarges until the fusion occurs, obtaining a more compact matrix with less channels for AN release, which leads to the next stage of release mechanism. In the second stage, the fertilizer hydrogel exhibited a controlled release pattern characterized by the slowest AN release (144–504 h), following the zeroth-order mechanism. In this stage, the significant reduction of AN release rate (*k*_0_ = 0.0023–0.0027) leads to the sustained presence of AN until the fertilizer hydrogel initiates its degradation. In the third stage, also called final release (720–1540 h), the polymeric network of hydrogel underwent degradation during its prolonged incubation, especially in soils. In this context, the A-CNFs predominantly diminished, and the polymeric matrix fragmented into oligomeric components. This transformation exposed larger pores, facilitating AN release, which was previously confined within the dense hydrogel matrix. Consequently, the Higuchi model governed this release stage, characterized by a new swifter release rate (*k_H_* = 0.0281–0.0268). The inherent properties of soil notably decelerated the rate of AN release across all release stages and pH levels. Compared to the buffer medium, it resulted in a more consistent and stable AN release. Figure 7e directly depicts the greenhouse pot experiment involved in the fertilizer hydrogel in an experimental agricultural farm.

Shang et al. [85] developed a temperature-responsive SRFH. Notably, the pore structure can offer appropriate sites for loading urea. The release mechanism of urea from hydrogels has been investigated through its release kinetics. The diffusion index (*n*) of the Korsmeyer–Peppas model reveals that *n* falls within the range of 0.43 to 0.85 when the temperature is below the lower critical solution temperature (LCST, 25 °C). This range signifies that the urea release from hydrogel follows a non-Fickian diffusion pattern, indicating an anomalous diffusion mechanism. In this period, the hydrogel needs to absorb water from the soil, which will permeate the hydrogel chains to facilitate nutrient dissolution. Subsequently, the release process commences once the polymer chain achieves relaxation. Specifically, 80% cumulative release within 12 h can be ascribed to the swelling of hydrogel at lower temperatures. In this state, the molecular chains within the network become entangled, thereby impeding the diffusion and release of fertilizer through the pores. When the temperature is above the LCST, it follows that *n* < 0.43, indicating that the urea release follows Fickian diffusion, which is controlled by the concentration gradient. At this point, urea in the hydrogel rapidly diffuses across diverse concentration gradients within and outside the system. It leads to an impressive cumulative release up to 98% from the hydrogel within the initial 6 h. This phenomenon could stem from the contraction of molecular chains in the hydrogel network, and the disruption of interactions between the fertilizer and the hydrogel. These factors improve the release of urea from the hydrogel. Consequently, this hydrogel can effectively modulate the release of loaded urea through the temperature stimuli.

Guo et al. [30] prepared a novel hydrogel MIL-100(Fe)@CNF-SA, which is involved in the fusion of MOFs with CNF and SA, acting as a carrier for urea. The urea-release mechanism from MIL-100(Fe)@CNF-SA was explored in depth, and the Higuchi model was selected as the most suitable model based on its high correlation coefficient. Specifically, due to its abundant carboxyl groups, the hydrogel after burying in soil starts adsorbing water and undergoes swelling gradually, leading to the expansions of the 3D network structure and volume of hydrogel. At this point, the free water enters the interior of the hydrogel. The urea molecule dissolves, and slowly diffuses out of the network pores through the dynamic exchange of free water. Then, the urea release can be slowed by the internal tortuous paths of hydrogels imparted by MIL-100(Fe), thus decreasing the release rate of fertilizer hydrogel. Finally, the extension of the hydrogel network slows down, which is accompanied by the saturation of hydrogel, leading to a reduction in urea release.

In summary, the slow-releasing capability of SRFHs offers outstanding water-retention performance, extending the efficacy of fertilizers, and decreasing the need for frequent applications. Furthermore, cellulose and its derivatives can contribute to the biodegradability of SRFHs, mitigating environmental concerns due to excessive fertilizer, and the promoting sustainable agricultural development.

## 5. Stimulus-Responsive Cellulose Hydrogels Serving as Slow-Release Fertilizers

Crops require different nutrients during various growth stages under different temperature conditions. Conventional hydrogels are insensitive to environmental conditions, and their swelling rate cannot change with external conditions [7,94]. Therefore, stimulus-responsive hydrogels emerged. These innovative hydrogels are able to detect subtle physicochemical changes in the external environment. After being triggered by external changes, such as pH, temperature, light, electricity, magnetism, force, etc., the stimulus-responsive hydrogels autonomously undergo stress responses [96]. Specifically, their structural properties, water absorption, solubility and other physicochemical traits will be in response to environmental variations.

Embedding fertilizer within stimulus-responsive cellulose hydrogels (so-called stimulus-responsive cellulose fertilizer hydrogels) can adjust the release behavior of fertilizer based on the fluctuations in surrounding soil. Moreover, it can deliver fertilizer precisely according to the plant’s nutrient demands in the right quantity at the optimal time [3]. Stimulus-responsive cellulose hydrogels not only compensate for the limitations of traditional fertilizers (e.g., insensitivity), but also provide a practical strategy for modern agriculture. This review provided a brief summary of stimulus-responsive cellulose fertilizer hydrogels as shown in Table 2. Predictably, they would achieve the slow-release of fertilizer, reduce fertilizer pollution, and minimize water resource wastage.

### 5.1. pH-Responsive Cellulose Fertilizer Hydrogels

A pH-responsive hydrogel is a type of polymer which will experience a phase transition (e.g., conformation, water absorption, solubility, volume, mechanical property, etc.) according to the pH change in its surroundings. Typically, these hydrogels contain ionizable acidic or alkaline groups. Broadly, pH-responsive hydrogels can be categorized into anionic (alkaline), cationic (acidic), and amphoteric hydrogels [3]. When the pH in the vicinity of hydrogel reaches a certain threshold, its protonation of ionizable groups occurs, which will lead to the hydrogen bonding and the electrostatic interaction among the molecular chains. Meanwhile, the concentration and type of ionic species change inside and outside the cellulose hydrogel simultaneously, which will cause a variation in osmotic pressure. As a consequence, the hydrogel begins to swell or shrink, exhibiting a macroscopic change in volume. In the exploration of SRF, pH-responsive cellulose hydrogels have attracted particular attention, because soil pH variation is very common. Specifically, the soil is slightly acidic, while dry soil is in an alkaline state in the alternating wetting and drying (AWD) cycle. Furthermore, soil in different geographical regions exhibits various pH values. The pH-responsive cellulose hydrogels can undergo volume and release behavior shifts in response to the pH variation, suggesting their potential in the field of controlled fertilizer release [15].

**Figure 8:**
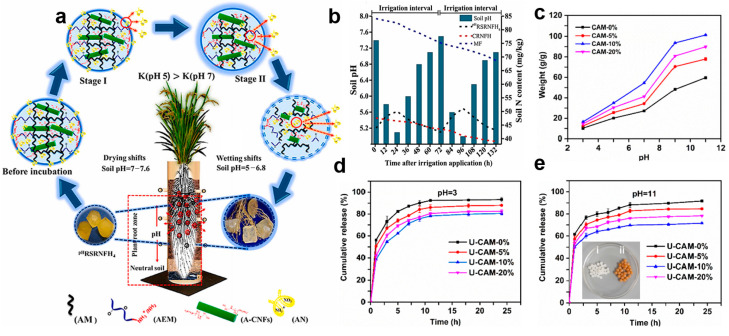
pH-responsive cellulose-based SRFH: (**a**) AN release of ^pH^RSRNFH varied at different pH, (**b**) N content and pH variations of soil containing ^pH^RSRNFH and other samples during ADW irrigation application. Reprinted with permission from ref. [3]. Copyright 2022 Elsevier Ltd. (**c**) CAMs display different solubilization capacities depending on pH variations. (**d**,**e**) Cumulative urea release of U-CAMs at a pH of 3 and 11. Reprinted with permission from ref. [15]. Copyright 2021 Elsevier Ltd.

Shaghaleh et al. [3] prepared a pH-responsive slow-release N fertilizer hydrogel (^pH^RSRNFH) based on A-CNF and poly(acrylamide-co-aminoethyl methacrylate hydrochloride) (PAM-PAEM) through direct AN fertilizer encapsulation. The pH range of neutral soil under AWD irrigation ranged between acidic/neutral and alkaline conditions. After culturation in buffer and soil for 58 and 65 days, the AN release of ^pH^RSRNFH was 3.00 and 2.69 mg·day^−1^ at a pH of 5.5, and 0.92 and 0.55 mg·day^−1^ at a pH of 7.4 (Figure 8b). The results showed that products had the greatest solubilization, water uptake and AN release at a low pH range of the surroundings. At this point, the plants have the most demand for N fertilizer. This is because the plant root system was activated by the increasing soil moisture during the wetting variation. In contrast, the lowest and slowest AN release was observed in dry soil in neutral and alkaline conditions, which is associated with the low nutrient demand of the plant root system. This mode of fertilizer application enables the necessary management of N fertilizer in accordance with plant demand. Therefore, the adoption of this innovative, pH-responsive and continuous AN delivery system certainly will contribute to sustainable development of agriculture, which offers a high-quality approach to N/water management, ensuring that the optimal N application rates are matched with suitable water-holding capacity and biodegradation rates.

The practical applications of cellulose hydrogels in agricultural scenarios and irrigation are intermittent. Therefore, we anticipate them to exhibit a responsive property to external stimuli, as well as repeatable shrinking and swelling capabilities. Wang et al. [15] designed the pH-responsive CNF/SA/MOF hydrogels (CAMs) with an impressive reusability. Specifically, the CAMs exhibited significant shrinkage in acidic conditions, which means the CAMs possess high absorption and substantial swelling properties in neutral and alkaline conditions. The results showed that the expansion of CAMs after loading urea (U-CAM) coincided with the gradual release of urea. Compared with other products, U-CAM demonstrated excellent swelling performance in an alkaline environment (Figure 8c). To explore its practical applications, the urea release from U-CAM was also investigated at pH = 3, 11. In Figure 8d, the cumulative release of CAM after loading 10% urea (U-CAM-10%) was the lowest, displaying a better slow-release performance. Moreover, its cumulative release was lower at a pH of 11 than at a pH of 3 (Figure 8e). The urea release of U-CAM-10% is satisfactory at a pH of 11, demonstrating that U-CAM is well suited for use in alkaline environments. As expected, U-CAM-10% exhibited the best performance in water adsorption even after three cycles of test at a pH of 11. In terms of reusability, CAM exhibited the ability to shrink in acidic environments, and swell in alkaline conditions, further proving its favorable pH-responsive behavior and repeatable shrinking and swelling capacities. Overall, the enhanced performance of CAMs in alkaline environments further indicates their potential in semiarid and arid regions.

### 5.2. Temperature-Responsive Cellulose Fertilizer Hydrogels

As a crucial and frequently encountered environmental factor, temperature can be readily controlled, and exerts a substantial influence on crop growth. Temperature-responsive cellulose hydrogels exhibit unique sensitivity to temperature variations such as volume, water absorption, swelling, light transmittance and structural properties [97]. They contain specific proportions of hydrophilic and hydrophobic groups, the hydrogen bonding of which can be influenced by the temperature variation, deciding the hydrophilicity or hydrophobicity of the product. The critical phase transition temperature refers to the temperature when a thermosensitive hydrogel shifts from shrinking to swelling or from swelling to shrinking [98]. Temperature-responsive hydrogels are typically classified into two categories: lower critical solution temperature (LCST) and upper critical solution temperature (UCST) types of hydrogels. LCST-type hydrogels generally possess both hydrophilic and hydrophobic groups [99]. For LCST-type hydrogels, when the temperature is higher than LCST, the interaction between hydrophilic and hydrophobic groups weakens, and the hydrophobic groups become dominant. This results in the structural instability of the hydrogel, leading to its absorption loss and shrinkage [100]. Conversely, when the temperature is lower than LCST, the hydration within the hydrogel intensifies, resulting in the swelling of hydrogel. This temperature-responsive behavior is also referred to as a negative temperature response [101]. For UCST-type hydrogels, when the temperature is lower than UCST, the hydrophobic groups dominate, leading to hydrogel shrinkage. When the temperature is higher than UCST, the hydrophilic groups become dominant, triggering the structure and performance variations, which results in the water absorption and expansion of the hydrogel, leading a macroscopic swelling appearance. Apparently, such temperature-responsive behavior is referred to as a positive temperature response [102].

**Figure 9:**
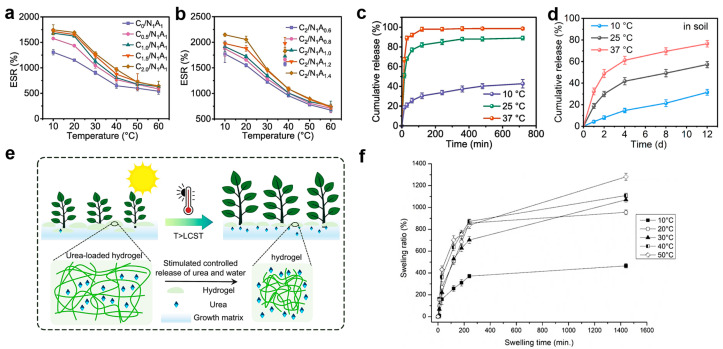
Temperature-responsive SRFH of CMC/poly(NVCL-co-AM): (**a**,**b**) ESR at different temperatures; cumulative urea release in (**c**) water and (**d**) soil; (**e**) temperature-response schematic. Reprinted with permission from ref. [85]. Copyright 2023 Elsevier Ltd. (**f**) Swelling ratio of hydrogel H5CA immersing in distilled water at different temperatures. Reprinted with permission from ref. [29]. Copyright 2021 MDPI Ltd.

Shang et al. [85] developed the temperature-responsive hydrogels by N-vinylcaprolactam (NVCL) aqueous dispersion through the emulsification of CMC and acrylamide (AM). Additionally, urea was loaded as a fertilizer into the hydrogels (Figure 9a–e). As the hydrophilic groups increase, the solubility of hydrogel at 20 °C can reach 2056% (Figure 9a,b). All hydrogels display a common pattern: elevated ESR at low temperature and reduced ESR at high temperature. The trend can be explained by the presence of temperature-sensitive PNVCL in hydrogel, which exhibits an extended state when the ambient temperature is below the LCST. When the hydrogel was heated above the LCST (25 °C), its PNVCL segment will undergo curling. This transition predominantly exposes the hydrophobic groups, and promotes the increasing hydrogen bonding within the polymer segments, hindering the transport of water molecules and resulting in the reduced ESR. In Figure 9c, the cumulative fertilizer release of the product below the LCST (over 12 h) reaches 80%. At temperatures of 37 °C, the hydrogel exhibited a more pronounced release behavior, reaching a cumulative release up to 98% within the initial 6 h (Figure 9c). A similar behavior was observed in the soil (Figure 9d). This proves that the urea release of hydrogel was effectively controlled through the temperature stimulation (Figure 9e).

Durpekova et al. [29] developed a novel hydrogel by crosslinking cellulose derivatives (HEC and CMC) with citric acid (CA, 15 wt%). The hydrogel H15CA that was integrated into the soil notably exhibited a higher water retention, which surpassed the original soil by nearly 30%. Compared with hydrogel H5CA (5 wt% CA), the urea and KNO_3_ released more slowly from hydrogel H15CA. Figure 9f demonstrates the relationship between the temperature variation and the swelling capacity of hydrogel. At a higher temperature (50 °C), a significant increase in the swelling ratio was observed within the initial few hours, followed by a gradual rise. After exposure to the media for 24 h, it reached the equilibrium in swelling. This illustrates that the best swelling capacity appeared at the highest temperatures, which might be attributed to the thermal expansion of the hydrogel network and the disruption of hydrogen bonding between polymer molecules. In contrast, the least water uptake was observed when it was immersed in the distilled water at a low temperature. Under a low-temperature condition (10 °C), its water uptake speed in the swelling process was slow, the swelling ratio of which was stabilized after 3 h.

### 5.3. Salt-Responsive Cellulose Fertilizer Hydrogels

Saline soils are not suitable for growing crops, and some areas even cannot support any plant growth. In China, saline and alkaline land is widely distributed in the north-eastern, northern and north-western regions, with a soil area of about 340,000 km^2^, of which 124,000 km^2^ can be used for agricultural production after improvement [103]. Songnen Plain is the main production area for grain and livestock in Northeast China [104,105]. However, the farmers in this region avoid irrigating their land because of the high salinity and mineralization of the local water. The main salts found in Songnen Plain are sodium carbonate (Na_2_CO_3_) and sodium bicarbonate (NaHCO_3_) [106]. The population of Northeast China continues to grow, but the amount of land available for cultivation decreases dramatically. Factors such as the new crown pneumonia and the Russian–Ukrainian conflict increase the cost of food production, threatening the global food security seriously. Therefore, saline soils have been considered as a potentially valuable land resource [107]. In arid inland areas, over-irrigation not only wastes the water, but also causes fertilizer loss, soil salinization and waterlogging. Currently, the fertilizer and water loss are significant issues in agricultural research [108]. Fertilizer retention agents are of high cost, have a difficult degradation and decay by mildew, and have a complicated operation and poor water absorption. Therefore, it is an important goal to develop novel fertilizer adsorbents that are low cost, salt resistant, water absorbent and available under saline conditions in the future.

**Figure 10:**
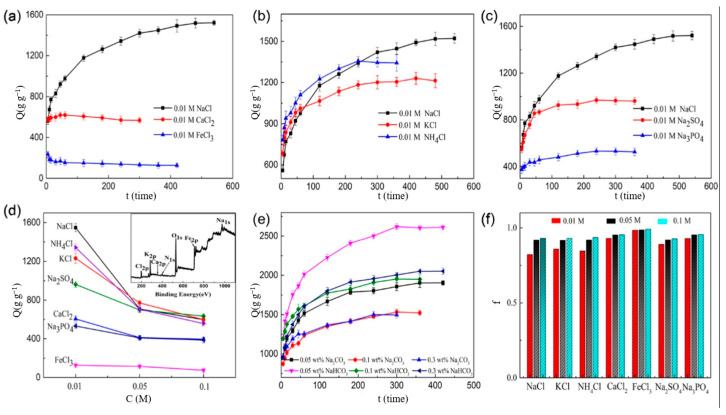
Salt-responsive cellulose SRFH of CFM: its swelling capacity in different saline solutions. (**a**) Cations with same valence; (**b**) cationic solutions; (**c**) anionic solutions; (**d**) equilibrium swelling under different salt concentrations, (Insert in (**d**) was component analysis); (**e**) equilibrium swelling in a Na_2_CO_3_ (or NaHCO_3_) solution; (**f**) salt sensitivity factor of CFM for various salts. (*Q* represents adsorption capacity; *C* represents concentration of solution; *f* represents sensitivity factor). Reprinted with permission from ref. [106]. Copyright 2019 Elsevier Ltd.

Qi et al. [106] synthesized the carboxymethyl cellulose fertilizer microspheres (CFM) with uniform pore structure, high porosity, good biodegradable capability and excellent fertilizer absorbency. Figure 10 illustrates the impact of saline solution on the water absorption of CFM. The absorption capacity of CFM varied based on the cations in saline solution, and the sequence from high to low is Na^+^, Ca^2+^ and Fe^3+^ (Figure 10a). Then, the water absorption of CFM was investigated in the saline solution containing various anions. From Figure 10b, the water absorption of CFM in the solution containing the polyatomic monovalent cation (NH^4+^) was lower than the monovalent cations (K^+^, Na^+^). This is because NH^4+^ may have reacted with –OH on the CFM chain to form ammonia, resulting in a denser internal structure of CFM and a decrease in water absorption capacity. The water absorbency of CFM in anionic solution following the order from highest to lowest is Cl^−^, SO_4_^2−^ and PO_4_^3−^ (Figure 10c). Finally, the water absorption of CFM in NaCl, KCl, NH_4_Cl, CaCl_2_, FeCl_3_, Na_2_SO_4_ and Na_3_PO_4_ solution was determined (Figure 10d–f). The water-absorption capacity of CFM decreases with the increasing salt concentration due to the osmotic pressure difference of the 3D network inside and outside the CFM. A smaller difference in osmotic pressure means that less solution enters the CFM. Since the salt concentration increases, the osmotic pressure difference decreases, the network structure of CFM shrinks, and its water-absorption capacity decreases [106]. The water absorptions of CFM in NaHCO_3_ and Na_2_CO_3_ solution were 1532 g·g^−1^ and 1496 g·g^−1^, respectively. In conclusion, CFM has good salt tolerance and responsiveness, which enhances the soil quality and crop yields, offering the potential to improve the saline soils in Songnen Plain.

Li et al. [109] developed a novel hydrogel based on wheat straw cellulose (WSC) with IPNs for the slow release of nitrogen and phosphorus nutrients. They investigated the swelling kinetics and fertilizer-release behavior of product in various saline solutions. Its water absorbency in NaCl, KCl and Na_2_SO_4_ solutions increased as the soaking time extended, and reached a swelling equilibrium within 45 min. However, the water absorbency of product in CaCl_2_ solution peaked initially, and then decreased to a specific value. Its swelling capacity in saline solution in order from largest to smallest is NaCl, KCl, Na_2_SO_4_ and CaCl_2_ solution (10 mmol/L), and its water absorbency towards different cation influence in order from smallest to largest is Ca^2+^, K^+^ and Na^+^. Notably, its total release amount and rate of fertilizer in saline solution were ranked from largest to smallest as NaCl, KCl and CaCl_2_. Meanwhile, its fertilizer release towards different cation influence followed the order from smallest to largest is Ca^2+^, K^+^ and Na^+^. The fertilizer release behavior of product is similar to its water-absorption behavior. These findings indicated that the swelling capacity of product was better under neutral condition. The cations and anions affecting the swelling capacity of product in order from largest to smallest is Na^+^, K^+^, Ca^2+^ and Cl^−^. The sensitivity of product towards the saline solution suggests its potential application in saline soils.

### 5.4. Multi-Responsive Cellulose Fertilizer Hydrogels

During actual crop growth, cellulose hydrogels in the soil will encounter a variety of conditions. Multi-responsive cellulose hydrogel is a versatile material designed to react to a spectrum of stimuli such as temperature, pH, salt concentration, light, etc. Typically, such a hydrogel consists of a combination of polymers such as PVA and PAA, which possess multiple functional groups (e.g., photosensitive groups, acid and base groups) [110]. Lin et al. [31] developed an MC hydrogel through the synergistic combination of TEMPO-oxidized CNFs and NVCL via free-radical polymerization. Notably, NVCL and CNF exhibited temperature and pH responsiveness, respectively. Even on the 30th day, MC-10% hydrogel still maintained a notable water retention (22.78%) in the soil, consistently demonstrating the reproducible shrinking and swelling traits in diverse response scenarios.

**Figure 11:**
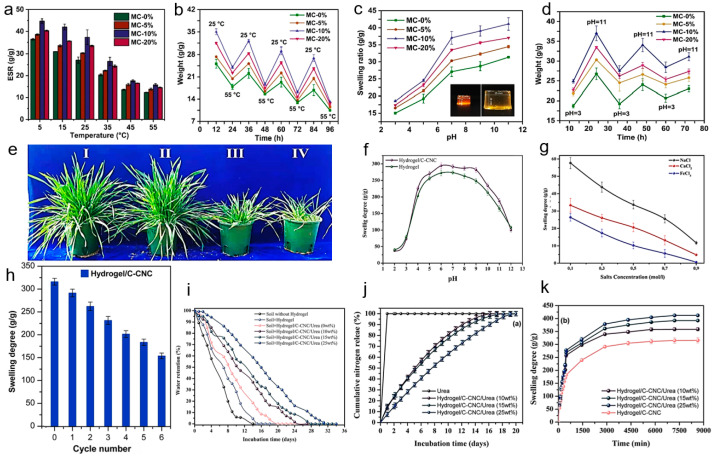
Multi-responsive cellulose SRFH MC: (**a**,**b**) ESR and swelling capacity at different temperatures; (**c**,**d**) swelling capacity at different temperatures; (**e**) practical photograph of wheat containing (I) MC-10%, (II) MC-0%, (III) pure urea and (IV) nothing. Reprinted with permission from ref. [111]. Copyright 2021 Elsevier Ltd. (**f**) pH effect on the swelling degree of hydrogel samples. (**g**) Water absorbency of hydrogel in saline solution. (**h**) Swelling property of hydrogel during consecutive swelling/drying cycles. (**i**–**k**) Water retention, cumulative nitrogen release and swelling degree of different hydrogels. Reprinted with permission from ref. [112]. Copyright 2022 Elsevier Ltd.

Figure 11a,b vividly illustrates the ESR decline of MC hydrogel at the increasing temperature. It indicated that MC hydrogels absorbed water more easily at a temperature lower than LCST (25 °C). However, the higher temperature (~30 °C) favored crop growth [111]. Thanks to the remarkable temperature-responsive features of MC hydrogel, its fertilizer release rate was elevated at a warmer temperature, which guaranteed the ample supply of nutrients for sustained crop growth. Additionally, the MC hydrogel exhibited good pH responsiveness. As shown in Figure 11c,d, the swelling ratio of hydrogel escalated as the pH ranged from 3 to 11. Meanwhile, the fertilizer release rate of MC hydrogel was reduced in the alkaline environment, which enhanced the fertilizer utilization efficiency in the environment for crop growth, especially at a pH of approximately 7.5. After the application of MC hydrogel in practice, the tillers and leaves increased, and the photosynthetic rate of wheat improved (Figure 11e). In summary, this temperature- and pH-responsive cellulose hydrogel contributed to crop growth, enhancing its good availability, serving as the SRF.

Since the global population is increasing and environmental pollution worsens, the area of arable land is diminishing. Moreover, factors including water use from rivers and lakes, underground irrigation, and fertilizer application contribute to the excessive accumulation of salt ions in the soil, resulting in soil salinization. This process further reduces the available agricultural land, and directly jeopardizes food security and crop production. Consequently, it is of immense significance to strategically develop a multi-responsive cellulose SRFH that possesses excellent biodegradability, water-retention capability, salt tolerance, and adaptation to the intricate and ever-changing environments in arid and semiarid regions. This endeavor aligns with the goals of the national agricultural strategy, which will certainly mitigate the issues for farming in these challenging regions.

Idrissi et al. [112] developed an innovative nanocomposite hydrogel with pH- and salt-responsive properties, which was achieved by conducting an in situ free-radical copolymerization involving SA, acrylic acid (AA) and AM in aqueous medium by utilizing N,N’-methylene bis-acrylamide (MBA) as a crosslinker and citric acid-functionalized cellulose nanocrystal (C-CNC) as nano-filler. As illustrated in Figure 11f, the hydrogels and their nanocomposites after loading urea displayed a minimal dissolution at a pH of 2, reached their maximum solubility at a pH of 6, and gradually decreased in their solubility with a further increase in pH (pH > 9). In addition, nanocomposite hydrogels were immersed in various saline media, including NaCl, CaCl_2_ and FeCl_3_. As depicted in Figure 11g, it is evident that the swelling phenomenon gradually diminishes with the increasing saline solution concentration. Furthermore, the absorption capacity of saline solution decreased with the increasing cation charge, following the order of Na^+^ > Ca^2+^ > Fe^3+^. In Figure 11h, it can be observed that the nanocomposite hydrogel with C-CNC exhibited a better reswelling capability than the hydrogel without C-CNC. The water retention experiment result showed that the soil containing Hyd/C-CNC could retain more water for a month (Figure 11i). The product prepared by the formulation containing 25 wt% of urea could release 86.37 ± 1.86% within 15 days (Figure 11j). The as-obtained nitrogen-rich superabsorbent exhibited enhanced water absorbency (412 ± 4 g/g), while also possessing the characteristic of slow-release nitrogen (Figure 11k). This finding suggests that the application of such a superabsorbent could alleviate the drought stress in arid and semiarid regions by improving the moisture retention of soil.

## 6. Summary and Prospect

Agriculture is the cornerstone of the national economy. An inappropriate or excessive use of traditional fertilizers leads to low nutrient utilization in crops, reducing crop yields, and increasing fertilization costs. In addition, nutrient leaching, denitrification, surface runoff and volatilization can cause adverse impacts to the environment, such as water and air pollution. It is particularly serious in the arid and semiarid regions with minimal precipitation, unobvious seasonal distribution, and low crop yields. The synergy effect between hydrogel and fertilizer enables the regulation of water and nutrients within a system. Research on environmentally friendly and efficient intelligent fertilizers is an essential prerequisite for the advancement of modern agriculture. This paper provides a comprehensive review of recent research progress concerning stimulus-responsive cellulose hydrogels in the applications of slow-release fertilizer. Although many significant advancements have been made, some challenges still demand further exploration and resolution.

(1) Food security serves as the fundamental pillar of national security. Although plants are the main source of cellulose, comprehensive assessments are still necessary to evaluate the toxicity of as-prepared cellulose hydrogels for SRF application. Additionally, it is imperative to develop eco-friendly organic solvents and crosslinking agents to ensure the nontoxicity of cellulose hydrogels throughout their entire life cycle.
(2) The biocompatibility and biodegradability of cellulose hydrogels are the key merits for SRF development. Generally, the biodegradation of cellulose in soil was once deemed advantageous, while it was also a potential drawback to a certain extent (e.g., susceptibility to microbes and enzymes), which presents a significant challenge and may not necessarily bring the anticipated benefits.
(3) The influences of external conditions on soil property, plant growth, and nutrient-release performance are still unclear. Despite the promising applications in SRF, the as-prepared stimulus-responsive cellulose hydrogels after loading fertilizers cannot control their nutrient release fully. This limitation hinders the synchronization of plant nutrient demands with the fertilizer release cycles. Therefore, the release mechanisms and rules of intelligent cellulose fertilizer hydrogels still need deep investigation.
(4) Particular emphasis should be placed on controlling hydrogel structure and composition, regulating release quantities, developing stimulus-responsive systems, and comprehending the impact of environmental sensitivity on swelling capacity and release kinetics. These efforts are necessary to cater to the diverse requirements for crop growth in varying environments, which will define the focal points and directions of future research.
(5) A combination method of cellulose hydrogel and fertilizer is of paramount importance for the slow-release performance of SRF. Many products prioritize the envelope or coating, but often neglect their combination. The solubilization and adsorption methods for loading nutrients as much as possible still remains limited, resulting in suboptimal fertilizer slow release. It is imperative to establish a comprehensive evaluation standard for SRF to provide robust guidance for industry advancement.

In conclusion, significant progress has been achieved in stimulus-responsive cellulose SRFHs, while it is crucial to transit these advancements into practical production technology for the agricultural field. Future endeavors should involve the utilization of advanced instruments, materials and technologies to enhance the preparation process of intelligent cellulose fertilizer hydrogels. Moreover, emphasis should be placed on recycling and maximizing the value of biomass resources, which indicates the immense potential of stimulus-responsive cellulose SRFHs for sustainable agricultural development and ecological environmental protection.

## Figures and Tables

**Table 1:** Release mechanism, breakthrough and challenge of cellulose SRFHs.

Release Mechanism	Breakthrough	Challenge	Ref.
Stage I: First-order release kinetics model $\frac{M_{t}}{M_{\infty }}=1-e^{tk_{1}}$	Sustained AN release over 90 days; N fertilizer delivery pattern of pH-stimulus cellulose hydrogel has been synchronized with plant demands in the critical growth periods; Root activation can be achieved under short-term pH variation of soils during wetting/drying irrigation intervals.	Water retention and holding capacity of hydrogel need more systematic evaluations; Deep investigation of nutritional requirements for crops at different growth stages; Detailed and systematic research on the sustained release mechanism of fertilizers is still scarce.	[3]
Stage II: Zero-order release kinetics model $\frac{M_{t}}{M_{\infty }}=k_{0}t$			
Stage III: Higuchi model $\frac{M_{t}}{M_{\infty }}=k_{H}t^{1/2}$			
Korsmeyer–Peppas model $\frac{M_{t}}{M_{\infty }}=kt^{n}$	Total release was 15% on the 1st day and no more than 75% on the 30th day; Hydrogels are in line with the SRF standard of the European Committee for Standardization;	Adjusting composition to increase the solubility, crosslinking degree, hydrophilicity, positive and negative charge of product.	[28]
	Hydrogel exhibited a swelling ratio exceeding 1400% at the optimal pH and temperature.	Study the composition of hydrogels after biodegradation; Ensure the nontoxicity, safety and reliability of hydrogels.	[29]
Higuchi model followed by Korsmeyer–Peppas model	Nanocomposite hydrogel maintained 17.36% of soil moisture within 30 days; High urea loading capacity of 1.47 g/g; Cumulative urea release capacity of 60% within 30 days.	Consideration should be given to the stimulus-responsive cellulose SRFHs for smart regulation of water and fertilizer.	[30]
	Hydrogels exhibited a better swelling-capacity (37 g/g), water-retention (22.78%) and slow-release performance (40.84%).	To achieve large-scale manufacture, the preparation process should be simplified;	[31]
	Hydrogel with high surface area (45.25 m^2^/g) showed high water adsorption (100 g/g); Lengthened period of totally losing the soil moisture by 18 days than pure soil.	Slow-release mechanisms should be studied in depth; Need economic evaluation of hydrogels, including raw materials, operation, fixed capital investment, etc.	[15]

**Table 2:** Nutrient loading species, combination ways, water-retention and holding ratio, and cumulative release of stimulus-responsive cellulose fertilizer hydrogels.

Stimuli	Nutrient	Combination Ways	Water-Retention and Holding Ratio	Swelling Capacity	Cumulative Release	Refs.
pH	Urea	In situ encapsulation	Water-holding ratio of 50% and complete loss of water after 30 d in soil.	Superior moisture content of 96.28%.	Only 50% of urea released in the 30th day.	[15]
	NPK		Water-retention ratio was above 80% at 60 d.	High equilibrium swelling capacity (60~70 g/g).	Cumulative release of NPK from hydrogels within 30 d was 67.90%, 70.78% and 71.12% in water; 64.52%, 53.72% and 64.08% in soil.	[28]
	N		-	-	AN release was 3.00 and 2.69 mg·day^−1^ at pH 5.5, while 0.92 and 0.55 mg·day^−1^ at pH 7.4 for 58 and 65 days in buffers and soil.	[3]
Temperature	Urea	In situ encapsulation	Water retention reached 50% in ~10 h.	Swelling ratio of hydrogels was 2056% at ~53 h.	When the temperature is lower than LCST, the cumulative release within 12 h was 80%, while it could reach 98% when the temperature was raised to 37 °C.	[85]
Temperature and pH	Urea	Solubilization and adsorption	Water retention increased to 77.53%, and remained at 22.78% at 30 d in soil.	Swelling capacity reached 37 g/g.	The cumulative release rates of MC-10% was 40.84% on 30 d.	[31]

## Data Availability

No new data were created or analyzed in this study.
